# Validation of the Greek Version of Social Appearance Anxiety Scale in Adolescents and Young Adults

**DOI:** 10.14806/ej.28.0.1027

**Published:** 2023-05-16

**Authors:** Triada Konstantina Papapanou, Flora Bacopoulou, Maria Michou, Christina Kanaka-Gantenbein, Dimitrios Vlachakis, George P. Chrousos, Christina Darviri

**Affiliations:** 1 Postgraduate Course of Stress Science and Health Promotion, School of Medicine, National and Kapodistrian University of Athens, Athens, Greece; 2 Center for Adolescent Medicine and UNESCO Chair on Adolescent Health Care, First Department of Pediatrics, School of Medicine, National and Kapodistrian University of Athens, Aghia Sophia Children’s Hospital, Athens, Greece; 3 Human Ecology Laboratory, Department of Home Economics and Ecology, Harokopio University, Kallithea, Athens, Greece; 4 Genetics Laboratory, Biotechnology Department, School of Applied Biology and Biotechnology, Agricultural University of Athens, Greece; 5 University Research Institute of Maternal and Child Health & Precision Medicine and UNESCO Chair on Adolescent Health Care, Medical School, National and Kapodistrian University of Athens, Aghia Sophia Children’s Hospital, Athens, Greece

## Abstract

Many people are worried about their social appearance. The fear of negative evaluation and judgment regarding one’s look in social circumstances is referred to as social appearance anxiety. Social appearance anxiety belongs to social anxiety. The aim of the present study was to validate the Social Appearance Anxiety Scale (SAAS) in the Greek language and to examine its psychometric properties. An online survey was conducted in a Greek population sample of adolescents and young adults aged 18 to 35 years. The survey instruments included the Social Appearance Anxiety Scale, the Social Physique Anxiety Scale (SPAS), 2 subscales of Multidimensional Body-Self Relations Questionnaire Appearance Scale (MBSRQ), the Appearance Schemas Inventory-Revised Scale (ASI-R) and the Depression Anxiety Stress Scale (DASS). A total of 429 respondents participated in this research. The statistical analysis showed that the Greek version of the SAAS has good psychometric properties. The internal consistency of questions within the SAAS was 0.942. Positive correlations were found between SAAS and SPAS, the overweight preoccupation subscale of MBSRQ, the ASI-R and the DASS, while negative correlations were observed between SAAS and the appearance evaluation subscale of MBSRQ and age. The results of this study suggest that the Greek version of SAAS can be used as a reliable and valid instrument in the Greek population

## Introduction

Social anxiety disorder consists of/includes several types of social fear, such as social interaction anxiety and fear of negative evaluation of the appearance. Most studies focus on social physique anxiety (Levinson and Rodebaugh, 2011). The meaning of Social Appearance Anxiety is a subtype of social anxiety and refers to the concern for the negative evaluation of appearance and the fear of rejection by others due to appearance (Hart *et al*., 2008; Claes *et al*., 2011). The anxiety about social appearance is caused by the idea that people cannot make a positive impression on other people (Leary and Kowalski, 1995) and is a result of their negative perception of the body and appearance. Social appearance anxiety is a kind of social anxiety and is defined as the anxiety and intensity that people experience when their external appearance is evaluated by other people (Haydar Şar, 2012). These people are therefore characterised by introversion, pessimism, insecurity, inadequacy in social relationships, keeping their distance and avoiding others and constantly wait for approval and acceptance from their environment. They also try to hide their body or parts of their body that they do not like. The social, academic and professional sectors can be negatively affected by social appearance anxiety (Baltaci *et al*., 2021). Social appearance anxiety includes a more complete concept of physical appearance, extending from the general physical characteristics associated with physiques such as height, weight and muscle structure to more personal characteristics such as skin, hair, face shape and size of features (Argon, 2014), therefore, the term broader as it includes both general appearance problems and physique concerns. (Hart *et al*., 2008; Levinson and Rodebaugh, 2011). Studies have found correlations between the social appearance anxiety and eating disorders, the fear of negative evaluation (Levinson and Rodebaugh, 2011; Brosof and Levinson, 2017; Hart *et al*., 2015; Levinson *et al*., 2013), the avoiding of social relationships, the feelings of loneliness and dependence on social media and the internet (Ayar *et al*., 2018;, Dogan and Çolak, 2016), the self-esteem, the body image (Claes *et al*., 2011; Demirel, 2019), the negative perception of body image (Hart et. al., 2008), dissatisfaction with body image (Baratelli, 2009; Boersma and Jarry, 2013; Dakanalis *et al*., 2016), body mass index and perfectionism (Levinson *et al*., 2013). Body image is a subjective experience and depends on how the person interprets oneself. How a person perceives his body shows how he perceives himself (Dixit and Luqman, 2018). Many are worried about some part of their body. Concerns about body image show up in childhood and are especially strong in adulthood (Quittkat *et al*., 2019). According to Sabiston *et al*., negative body image and physical dissatisfaction can lead to the presence of social appearance anxiety (Sabiston *et al*., 2007). Furthermore, body checking behaviors occur when someone control or monitor possible changes in weight or body shape (Shafran *et al*., 2004). According to Claes (2011), social appearance anxiety showed positive correlations with the negative body image and social anxiety. A study in German population found a positive correlation of the SAAS scale with measures of social anxiety and disorders related to body image, such as greater concern for diet, weight and body shape, control of the body (body checking) and avoiding focus on the body (body avoidance). A weak positive correlation with BMI was also found (Reichenberger *et al*., 2021).

There are some measures that are used for social anxiety such as the Social Physique Anxiety Scale (Hart *et al*., 1989), the Liebowitz Social Anxiety Scale (Liebowitz, 1987), and the Social Phobia Inventory (Argyrides *et al*., 2014). These scales are focused on more particular characteristics of appearance, hence questionnaires that assess more general characteristics of appearance are needed. The social appearance anxiety scale (SAAS) is a self -reported measure with 16 items designed by Hart *et al*. (Hart *et al*., 2008). This scale has been translated and validated in various languages such as Turkish (Doğan, 2010), Persian (Iranian) (Goodarzi *et al*., 2021), Italian (Dakanalis *et al*., 2016), German (Reichenberger *et al*., 2021), Portuguese (Donofre *et al*., 2021), with good psychometric properties. The purpose of this research was to validate the SAAS in the Greek language and to assess its psychometric properties in a sample of adolescents and young adults aged 18–35 years, in Greece.

## Materials, Methodologies and Techniques

### Translation procedure

In the present study and validation of scale, the methodology of translation and intercultural adaptation was followed. The instrument was freely available, and no special permission for its use and validation was needed. Two independent individuals, who were Greek native speakers with an advanced level of English/very good knowledge of English, translated the scale from English to Greek (forward translation). Comparison of the two translations resulted in the first version of the scale in Greek and then one bilingual individual translated it from Greek into English (backward translation). The back-translation was compared with the original scale to record any disagreements. Subsequently, the Greek version was administered to 16 people for a test-retest to identify unclear points, to make the necessary corrections, and to determine its final version.

### Participants and procedures

The questionnaires were distributed online using Google Forms through various social media platforms such as Facebook, Instagram, Viber, and e-mails. Study participants were undergraduate or postgraduate students aged 18–35 years, employees or unemployed who were able to read and write in the Greek language. People with a diagnosis of severe mental disorder under medication were excluded. Data were collected online from the 2nd of March until the 30th of June 2022.

### Ethical considerations

The study’s protocol was approved by the scientific committee of the “Science of Stress and Health Promotion” Master’s Program and the Bioethics Committee of the Medical School of the National and Kapodistrian University of Athens (protocol number 611/25.02.2022). Participants were fully informed about the aims of the study through a brief text of the study’s protocol and were asked to tick a square to indicate their consent to participate in the research. The submission of their response was considered as online consent. Participants were also informed that they could leave the research whenever they wanted. The completion of the questionnaires was anonymous and voluntary, with the ability of withdrawal and termination at any time, without any consequences. The completion time was approximately 15 minutes.

### Measures

#### Sociodemographic Questionnaire

Participants completed information such as sex, age, height, weight, marital status, educational level, work status, social media use and time of use (hours per day).

#### Social Appearance Anxiety Scale (SAAS)

It is a self-report scale designed by Hart *et al*., in 2008, which consists of 16 items. It measures the anxiety that is created when someone is negatively evaluated and judged by others because of one’s overall appearance, including body shape. It is used a 5-point Likert scale with a range from 1 (not at all) to 5 (extremely). The first item is reverse-coded. The range score is between 16 to 80. Higher scores in this scale mean high level of social appearance anxiety. It consists of a single factor and has no subscales. Cronbach’s coefficient was reported as 0.94, 0.95, 0.94 for the three samples in the original study. An example of the items includes “I feel comfortable with the way I appear to others” (Hart *et al*., 2008).

#### Social Physique Anxiety Scale (SPAS)

This is a 12-item scale, without subscales, that measures the level of anxiety that a person experiences when realises that others evaluate or may negatively evaluate the physique, the anxiety about physical appearance, when the person is in an environment where he considers that his/her body is subject to evaluation by others. The scale is focused on physique-related issues such as body fat, muscle tone, body proportions and does not include other related areas of appearance anxiety. Each item is answered on a 5-point Likert scale ranging from 1 (not at all) to 5 (too much) (Hart *et al*., 1989). The reliability index of the Cronbach a was 0.85 and the reliability coefficient of examination-re-examination was r=0.84. The questionnaire has been translated and validated in the Greek language (Psychountaki *et al*., 2004).

#### Multidimensional Body-Self Relations Questionnaire Appearance Scale (MBRSQ-AS)

This is a 34-item questionnaire that evaluate attitudes / beliefs for body image. Every item is rated on a 5-point Likert scale ranging from 1 (definitely disagree) to 5 (definitely agree). This edition is a short version of a widely used 69-item questionnaire. It consists of 5 subscales but in our research will be used 2 of the 5 subscales.

#### Appearance evaluation subscale (7 items)

This subscale evaluates feelings of physical attractiveness or unattractiveness. High scores indicate greater positive feelings and satisfaction with the appearance of the body (Cash, 2000b).

#### Overweight Preoccupation subscale (4 items)

This subscale assesses one’s anxiety about weight, weight control or vigilance, dieting and nutritional restraint (Cash, 2000b). It has been validated in the Greek language with good psychometric properties (Argyrides *et al*., 2013).

#### Appearance Schemas Inventory-Revised (ASI-R)

The first version consists of 14 items, but after some changes the final version consists of 20 items (revised ASI-R), which include 2 subscales. Items are answered on a five-point scale ranging from 1 (strongly disagree) to 5 (strongly agree). This scale evaluates the beliefs about the importance meaning and effects of appearance on one’s life. Also, it evaluates the malfunctioning shape of the body image (Cash and Labarge, 1996). Self-Evaluative Salience (12 items)

This subscale refers to the way people believe that their own self-worth could be determined by their physical appearance.

#### Motivational Salience (8 items)

This subscale concerns an individual’s commitment with their appearance, such as grooming behaviors (Cash, 2003).

The ASI-R has been found to have good psychometric properties and it has been validated in the Greek Language (Kkeli and Argyrides, 2013).

#### Depression Anxiety Stress Scale – 21 items (DASS-21)

It is a self-report questionnaire and consists of three self-administered scales, which designed to measure depression, anxiety and stress. Each of the subscales consists of 7 questions, 21 total questions. A 4-point Likert scale is used from 0 (did not apply to me at all) to 3 (applied to me too much or most of the time). It has been validated in the Greek language and is considered a reliable and valid tool for measuring depression, anxiety and stress in non-clinical population (Pezirkianidis, 2018)

### Statistical analysis

Data are presented as frequencies (%) for the categorical variables and means, standard deviations (SD), median and interquartile range (IQR) for continuous variables. The sample adequacy and correlation between items were assessed with the Kaiser-Meyer-Olkin (KMO) statistic and Barlett’s Test of Sphericity. The factors identification of the SAAS was done by the Principal component analysis (PCA). Cronbach’s alpha values were calculated to examine the internal consistency of the questionnaire. Correlations between the SAAS and other measurements of the study were calculated. Due to the fact that the normality of data distribution was violated, non-parametric Spearman’s rho coefficient was conducted to examine correlations between quantitative variables and the SAAS. Non-parametric Mann-Whitney U and Kruskal-Wallis tests were used to evaluate between-group differences. Statistical analyses were performed using IBM SPSS version 24.0 for Windows.

## Results

The sample consisted of 429 participants from the general adolescent and young adult population in Greece. Participants’ sociodemographic characteristics and measurements are presented in Table 1. The median age was 25 years (IQR=6). Most participants were females (69.2%), unmarried (91.6%), with a Bachelor’s Degree (41.7%) or high school education (34.5%) and most of them were either university students (35.2%) or private employees (29.8%).

Table 2 shows the results of the Principal Component Analysis (PCA) of the 16 items with orthogonal rotation (varimax). The adequacy of the sample was examined using the Kaiser-Meyer-Olkin test and the KMO coefficient was 0.948. Barlett’s test of sphericity χ2(120) = 4795.374, p<0.0001, showed that correlations between items were sufficiently large to perform PCA. Only one component had eigenvalue >1 which explained 55.26% of the total variance and all factor loadings were >0.45. The factor load of the items was within the range of 0.456–0.874.

Table 3 presents descriptive statistics for the SAAS, such as range, mean, standard deviation, item - total correlation and Cronbach’s alpha coefficient. The score range of the scale was 16–80, the mean (SD) was 33.92 (13.76). The reliability of the SAAS scale was investigated by internal consistency according to the Cronbach’s a. The value of the coefficient was 0.942.

The score of participants in the SAAS scale was correlated with other scales. As shown in Table 4, there were statistically significant correlations with all scales with p<0.0001. SAAS showed weak positive correlations with the subscale Motivational Salience of the ASI-R (r=0.208) and with the subscale Overweight Preoccupation (r=0.291) of the MBSRQ, moderate positive correlations with the subscale for the anxiety (r=0.404), the stress (r=0.442) and the depression (r=0.451) of the DASS, moderate to strong positive correlations with the SPAS (r=0.791) and the subscale of Self-Evaluative Salience of ASI-R (r=0.659). Furthermore, a negative correlation was found with the subscale Appearance Evaluation of the MBSRQ (r=−0.600). Likewise, there was a significant negative correlation between SAAS and the age of participants with r=−0.123 (p=0.001).

Table 5 presents associations between the SAAS and other study variables. There was statistically significant difference in SAAS according to sex, as women scored higher than men (p<0.0001). Also, unmarried participants scored higher than married participants (p=0.028). According to the job status, unemployed individuals scored higher than those who were public employees, private employees, freelancers or university students (p<0.0001).

## Discussion

The current study aimed to evaluate the validity and reliability, factor structure and psychometric properties of the Social Appearance Anxiety Scale in the Greek language. The SAAS in our study have demonstrated good internal consistency with Cronbach’s alpha= 0,942, which is consistent with the results of the initial research of Hart *et al*. (2008) (a1 = 0.94, a2 = 0.95, a3 = 0.94 for the 3 samples) and with validations in other languages (Reichenberger *et al*., 2021; Doğan, 2010; Goodarzi *et al*., 2021; Donofre *et al*., 2021).

In this study women had higher levels of social appearance anxiety than men. This finding has been confirmed by other researchers, who found that women had higher score of SAAS than men, and therefore more social appearance anxiety (Reichenberger *et al*., 2021; Dakanalis *et al*., 2016; Levinson and Rodebaugh, 2011). In the original research the scores of SAAS were not correlated with female sex (Hart et. al., 2008) and this finding was consistent with other studies in which participants’ SAAS scores did not differ considerably according to sex (Amil and Bozgeyikli, 2015; Şahin *et al*., 2013). In line with other studies, SAAS demonstrated a significant positive correlation with SPAS (Hart *et al*., 2008; Goodarzi *et al*., 2021). Additionally, in our study SAAS was positive correlated with measures such as overweight preoccupation, and negative correlated with the appearance evaluation, which is consistent with the results of another validation of the scale (Reichenberg *et al*., 2021) and the original study (Hart *et al*., 2008). Furthermore, the results of the validation showed a moderate positive correlation between the SAAS and the DASS. This finding is consistent with the results of previous studies. A study of Malaysian students found that those who had high levels of social appearance anxiety were more likely to have high scores on depressive symptoms (Kadir *et al*., 2014). In general, positive correlations of social appearance anxiety with depression, anxiety and stress have been found. It is observed that as social appearance anxiety increases, the levels of depression, anxiety and stress also increase (Çelik and Tolan, 2021). Furthermore, people who are worried about their appearance have high scores of depression, anxiety and stress (Öncü *et al*., 2013). In terms of predictive validity, the SAAS seemed to be the only prognostic factor of depression beyond the indicators of social anxiety and negative body image (Hart *et al*., 2008).

This study has some limitations. Firstly, it was not possible to perform a test-retest analysis to examine the consistency of the instrument over time due to anonymity. Secondly, the sample mainly consisted of women, limiting the generalisation of the findings. In future studies, it is important to investigate the validity of SAAS factors in different/other samples and to evaluate the convergent and divergent validity with additional measures. Future research could compare the levels of SAAS between psychiatric and non-psychiatric patients and use this tool in clinical populations with social anxiety disorder, eating disorders, physical dysmorphic disorder, orthorexia, other medical conditions that affect appearance such as dermatological diseases or deforming conditions, in younger adolescents and in older adults and the elderly.

In conclusion, the present study indicates that the Greek version of Social Appearance Anxiety Scale has good psychometric properties. The scale refers to the fear of being judged by others based on one’s physical appearance, such as body shape. The SAAS can be considered a reliable tool for measuring anxiety of adolescents and young adults in situations where the assessment/judgement of one’s appearance is possible.

## Figures and Tables

**Table 1.** Participants’ sociodemographic characteristics and measurements.

Sociodemographic characteristics N=429		Scales and subscales scores		
Sex N (%)		SPAS score	Median (IQR)	31.00 (13.00)
- Women	297 (69.2%)		Mean (SD)	31.87 (9.10)
- Men	132 (30.8%)			
Age Median (IQR)	25.00 (6.00)	MBSRQ score		
Mean (SD)	25.30 (4.62)	- Appearance evaluation	Median (IQR)	26.00 (7.00)
			Mean (SD)	25.18 (5.33)
Marital status N (%)				
- Married	36 (8.4%)	- Overweight preoccupational	Median (IQR)	9.00 (5.00)
- Unmarried	393 (91.6%)		Mean (SD)	9.54 (3.69)
Education level N (%)		ASI-R Score		
- Until High School	148 (34.5%)	-Self evaluative salience	Median (IQR)	35.00 (12.5)
- Higher Education Institution/Technological Educational	179 (41.7%)		Mean (SD)	34.80 (9.13)
Institute		- Motivational Salience	Median (IQR)	27.00 (8.00)
- College	17 (4%)		Mean (SD)	26.73 (5.60)
- MSc-PhD	85 (19.8%)			
Job Status N (%)		DASS Score		
- Public Employee	48 (11.2%)	-stress	Median (IQR)	8.00 (8.00)
- Private Employee	128 (29.8%)		Mean (SD)	8.40 (5.76)
- Freelance	49 (11.4%)			
- University Student	151 (35.2%)	-anxiety	Median (IQR)	4.00 (9.00)
- Unemployed	41 (9.6%)		Mean (SD)	5.83 (5.60)
- Other	12 (2.8%)			
		-depression	Median (IQR)	5.00 (9.00)
			Mean (SD)	6.79 (6.12)

**Table 2.** Rotated factor loadings of the principal components analysis (PCA) for 16 items of SAAS (N= 429).

Item	SAAS
1. I feel comfortable with the way I appear to others.	0.551
2. I feel nervous when having my picture taken.	0.456
3. I get tense when it is obvious people are looking at me.	0.502
4. I am concerned people won’t like me because of the way I look.	0.767
5. I worry that others talk about flaws in my appearance when I’m not around.	0.729
6. I am concerned people will find me unappealing because of my appearance.	0.830
7. I am afraid people find me unattractive.	0.818
8. I worry that my appearance will make life more difficult for me.	0.770
9. I am concerned that I have missed out on opportunities because of my appearance	0.727
10. I get nervous when talking to people because of the way I look.	0.743
11. I feel anxious when other people say something about my appearance.	0.773
12. I am frequently afraid that I won’t meet others’ standards of how I should look.	0.794
13. I worry people will judge the way I look negatively.	0.874
14. I am uncomfortable when I think others are noticing flaws in my appearance.	0.786
15. I worry that a romantic partner will/would leave me because of my appearance	0.774
16. I am concerned that people think I am not good looking.	0.850
Eigenvalues	8.842
% of Variance	55.26

**Table 3.** Range, mean, standard deviation (SD), item-total correlation and Cronbach’s alpha coefficient of the SAAS scale.

Scale	Range	Mean (SD)	Min-Max	Item-total correlations	Alpha of scale
SAAS	16–80	33.92 (13.76)	16–79		0.942
1. I feel comfortable with the way I appear to others.				0.502	
2. I feel nervous when having my picture taken.				0.424	
3. I get tense when it is obvious people are looking at me.				0.469	
4. I am concerned people won’t like me because of the way I look.				0.725	
5. I worry that others talk about flaws in my appearance when I’m not around.				0.681	
6. I am concerned people will find me unappealing because of my appearance.				0.791	
7. I am afraid people find me unattractive.				0.778	
8. I worry that my appearance will make life more difficult for me.				0.720	
9. I am concerned that I have missed out on opportunities because of my appearance				0.668	
10. I get nervous when talking to people because of the way I look.				0.697	
11. I feel anxious when other people say something about my appearance.				0.736	
12. I am frequently afraid that I won’t meet others’ standards of how I should look.				0.750	
13. I worry people will judge the way I look negatively.				0.846	
14. I am uncomfortable when I think others are noticing flaws in my appearance.				0.753	
15. I worry that a romantic partner will/would leave me because of my appearance				0.728	
16. I am concerned that people think I am not good looking.				0.814	

**Table 4.** Correlations (Spearman’s rho) between SASS and other study measurements.

	Age	SPAS	MBSRQ Appearance Evaluation	MBSRQ Overweight Preoccupation	ASIR Self Evaluative salience	ASIR Motivational Salience	DASS Stress	DASS Anxiety	DASS Depression
Spearman rho	−0.123	0.791	−0.600	0.291	0.659	0.208	0.442	0.404	0.451
p-value	0.001	<0.0001	<0.0001	<0.0001	<0.0001	<0.0001	<0.0001	<0.0001	<0.0001

**Table 5.** Associations between the SAAS scale and other study measurements.

Study measurements	Categories	SAAS Median (IQR) Mean (SD)
Sex Median (IQR) Mean (SD)	Males	25.00 (17.00)
		30.06 (13.16)
	Females	33.00 (18.00)
		35.63 (13.69)
	p-value	<0.0001
Education level Median (IQR) Mean (SD)	Until High School	31.00 (22.50)
		35.14 (15.76)
	Higher Education Institution/Technological Educational Institute	30.00 (20.00)
		33.22 (13.25)
	College	35.00 (16.50)
		35.53 (11.95)
	MSc-PhD	30.00 (16.00)
		32.93 (11.19)
	p-value	0.742
Marital status Median (IQR) Mean (SD)	Married	27.50 (16.25)
		29.83 (13.01)
	Unmarried	31.00 (19.00)
		34.29 (13.78)
	p-value	0.028
Job status Median (IQR) Mean (SD)	Public Employee*	28.50 (17.50)
		30.13 (10.05)
	Private Employee^	28.50 (19.00)
		32.50 (13.50)
	Freelance<	27.00 (13.00)
		29.98 (11.85)
	University Student>	34.00 (21.00)
		35.44 (14.54)
	Unemployed * ^ < > -	40.00 (18.50)
		42.54 (14.35)
	Other-	31.00 (18.00)
		31.67 (10.75)
	p-value	<0.0001

* ^ < > - indicate significant differences between the groups

## Key Points

- Questionnaires for social appearance anxiety in Greece are lacking.
- The Social Appearance Anxiety Scale (SAAS) measures the anxiety created when someone is negatively evaluated and judged by others because of one’s overall appearance.
- The Greek version of SAAS was validated in a sample of 429 adolescents and young adults.
- The Greek version of the SAAS demonstrated good psychometric properties and internal consistency.
